# Controlled attenuation parameters to assess liver steatosis in obese patients with polycystic ovary syndrome

**DOI:** 10.3389/fendo.2023.1241734

**Published:** 2023-08-31

**Authors:** Dongxu Wang, Nan Nan, Hao Bing, Bing He

**Affiliations:** ^1^ Department of Gastroenterology, Shengjing Hospital of China Medical University, Shenyang, China; ^2^ Department of Endocrinology, Shengjing Hospital of China Medical University, Shenyang, China

**Keywords:** polycystic ovary syndrome, non-alcoholic fatty liver disease, controlled attenuation parameter, hyperandrogenemia, testosterone

## Abstract

**Objectives:**

This study was performed to investigate the changes and influencing factors of liver controlled attenuation parameter (CAP) in obese patients with polycystic ovary syndrome (PCOS), and to determine the prevalence and risk factors of nonalcoholic fatty liver disease (NAFLD) in PCOS patients with obesity.

**Methods:**

Forty-one PCOS patients with obesity and twenty age- and body mass index (BMI)-matched control women without PCOS were enrolled in this study. General data, body composition, biochemical parameters, sex hormones, and liver CAP in the two groups were collected and compared. Liver CAP was measured using transient elastography.

**Results:**

NAFLD was more common in the Obese PCOS group than in the control group (75.61% vs. 45.00%, *P*=0.018). Compared to the control group, the obese PCOS group showed apparent increases in alanine transaminase (ALT), aspartate transaminase (AST), CAP, triglyceride (TG), total cholesterol (TC), low-density lipoprotein cholesterol (LDL-C), totle testosterone (TT), free androgen index (FAI), fasting insulin (FIns), and homeostasis model assessment-insulin resistance (HOMA-IR), along with lower high-density lipoprotein cholesterol (HDL-C) and sex hormone binding globulin (SHBG) levels. In addition, as shown by Spearman analysis, liver CAP in PCOS patients with obesity had a positive correlation with ALT, AST, TG, TT, FAI, FIns, and HOMA-IR, and a negative correlation with SHBG. Logistic regression analysis showed that TG, TT, FIns, and HOMA-IR were risk factors for NAFLD, while TT was an independent risk factor for NAFLD in PCOS patients with obesity.

**Conclusion:**

PCOS patients with obesity had a significantly higher prevalence of NAFLD. Furthermore, in PCOS patients with obesity, liver CAP was associated with disorders of lipid metabolism, insulin resistance, and hyperandrogenemia, with elevated testosterone levels being an independent risk factor for NAFLD in PCOS patients with obesity.

## 1 Introduction

Polycystic ovary syndrome (PCOS) is the most common reproductive, endocrine, and metabolic disorder among women of childbearing age. The prevalence of PCOS in adult Chinese women is 5.6% (1, 2). The most common clinical manifestations of PCOS include chronic anovulation, hirsutism or acne, menstrual cycle disorder, and infertility, which are closely related to obesity, insulin resistance (IR), and abnormal lipid metabolism (3). Up to 80% of PCOS patients are overweight or obese (4).

Recent studies have shown that the prevalence of non-alcoholic fatty liver disease (NAFLD) in PCOS patients is significantly higher than that in the normal population (5), and this phenomenon is more common in PCOS patients with obesity (6). NAFLD is defined as a liver fat accumulation ≥ 5% in patients in whom secondary causes, such as viral hepatitis, excessive drinking, drug-induced liver disease, autoimmune liver disease, and hereditary metabolic liver disease, can be excluded (7). Liver biopsy remains the gold standard for the diagnosis of NAFLD, but its invasive characteristics limit its clinical application (8). Ultrasonography, as a simple and convenient examination method, is the most commonly used screening method for NAFLD in the clinic; however, the screening results of ultrasonography are influenced by the subjective judgment of the examiners, and the sensitivity to the diagnosis of liver steatosis below 20% is obviously reduced (9). Therefore, a sensitive, non-subjective test is required to screen for NAFLD.

The Controlled Attenuation Parameter (CAP), which is based on the principle of instantaneous elastography, quantifies liver steatosis by measuring the attenuation of the ultrasound beam in direct correlation with liver fat content (10). High concordance between CAP and liver biopsy enables CAP to better reflect liver steatosis (11). FibroTouch, a third-generation liver transient elastography device (12), has been widely used in the diagnosis of NAFLD. Therefore, this study used FibroTouch to measure liver CAP in PCOS patients with obesity to explore the prevalence and risk factors of NAFLD in PCOS patients with obesity.

## 2 Materials and methods

### 2.1 Patients

Forty-one PCOS patients with obesity attending the endocrine clinic of the Shengjing Hospital of China Medical University from October 2020 to January 2022 were consecutively enrolled in the study. Twenty non-PCOS women matched for age and body mass index (BMI) were consecutively selected as control subjects during the same period. This study was audited by Medical Ethics Committee of Shengjing Hospital of China Medical University (Audite No. 2021PS647K), and informed consent was obtained from each patient.

### 2.2 Inclusion criteria and exclusion criteria

#### 2.2.1 Inclusion criteria

(i) Premenopausal women aged over 18 who were diagnosed with obese PCOS for the first time. (ii) PCOS was diagnosed following the Rotterdam Standard (13), for which at least two of the following three conditions must be met: clinical manifestations of hyperandrogenemia (HA) and/or HA; Rare ovulation or anovulation; Ovarian polycystic changes (unilateral or bilateral ovaries 2–9 mm, follicles ≥12); or ovarian volume ≥10 ml (ovarian volume=0.5×length×width×thickness). (iii) Obesity was defined as a BMI ≥ 28 kg/m2, in accordance with the BMI classification criteria in China (14).

#### 2.2.2 Exclusion criteria

(i) Women with other diseases that may cause HA or ovulation disorder, such as Cushing’s syndrome, thyroid diseases, congenital adrenal hyperplasia, hyperprolactinemia, androgen-secreting tumours of the adrenal gland or ovary, etc. (ii) Age under 18 years of age or postmenopausal. (iii) Previous clear diagnosis of PCOS. (iv) Chronic liver diseases, such as alcoholic liver disease (alcohol consumption ≥ 20 g per day), viral hepatitis, autoimmune liver disease, drug-induced liver disease, and hereditary metabolic liver disease, excluding patients with cardiopulmonary insufficiency, renal insufficiency, and malignant tumours. (v) A history of taking hepatoprotective drugs or drugs affecting glucose and lipid metabolism in the last 3 months.

### 2.3 CAP testing

FibroTouch (Haskell Medical Technology Co., Ltd., Wuxi, China), operated by two professionally trained and experienced operators, was blinded used to measure CAP of the PCOS patients and controls. First, the imaging site was selected with the machine’s B-ultrasound imaging probe, and the 7th, 8th, and 9th intercostal spaces between the right axillary front line and the axillary midline were selected as the detection area, while avoiding intrahepatic cysts, bile ducts, and great vessels, to confirm the clear structure of the imaged liver tissue. The probe coated with the coupling agent was close to the patient’s intercostal space at the positioning point and perpendicular to the skin surface. A small pressure was applied to ensure that the probe pressure was in a suitable area for measurement. At least 10 effective measurements were taken at the same measuring point, and the success rate was at least 75%. The relative deviation was less than 30%, the image required a straight A wave, uniform M wave layer, and normal elastogram waveform, and the CAP value (db/m) was obtained. NAFLD was defined as a CAP ≥ 244 db/m (15).

### 2.4 Measurement index

#### 2.4.1 General data measurement

After fasting for 12 hours, patients and controls took off their normal shoes and put on light clothes, and their height and weight were measured during a quiet resting state in the morning. Body mass index (BMI) was calculated as weight (kg) divided by height (m) squared.

#### 2.4.2 Body composition examination

Waist-to-hip ratio (WHR), body fat percentage, and visceral fat area (VFA) were measured using an InBody 770 scanner (Inbody Co., Seoul, Korea).

#### 2.4.3 Measurement of biochemical indexes and sex hormone levels

Fasting blood samples were collected from the antecubital vein.

Biochemical parameters were measured using an automated biochemical analyzer (Abbott Architect ci 16200, Abbott, USA). Alanine aminotransferase (ALT) and aspartate aminotransferase (AST) were measured using NADH assays. Triglyceride (TG) levels were measured using deionization and enzymatic methods. Total cholesterol (TC) was measured using the cholesterol oxidase method. Low-density lipoprotein cholesterol (LDL-C) and high-density lipoprotein cholesterol (HDL-C) levels were measured using chemically modified enzymatic methods. Fasting blood glucose (FBG) levels were measured using the hexokinase/glucose-6-phosphate dehydrogenase method. Fasting insulin (FIns) levels were measured using the chemiluminescence (double antibody sandwich) method. Homeostasis model assessment-insulin resistance (HOMA-IR) was calculated using the following formula: HOMA-IR = FBG (mmol/L) ×FIns (mU/L)/22.5 (16). Fasting hyperinsulinemia and IR was defined by ≥95th percentile of our healthy reference population: that is, FIns > 11.8 mU/L, and HOMA-IR > 2.54 (17).

Sex hormone indicators were measured using an automated immunoassay (UnicelDxl800, Beckman Coulter, USA). The total testosterone (TT) level was assessed using an electrochemiluminescent immunoassay. The level of sex hormone-binding globulin (SHBG) was assessed using immunochemical luminescence assay. Free androgen index (FAI) was calculated using the following formula: FAI=TT (nmol/L) ×100/SHBG (nmol/L) (18).

### 2.5 Sample size estimation

For sample size calculation, a formula proposed by Charan et al. for cross-sectional studies was applied (19). The available data on CAP for PCOS and controls were used (287.3 ± 61.5db/m and 235 ± 39 db/m) (20), and with alpha set at 0.05, power at 90%, margin absolute error at 10%, and a sample size ratio of 2:1 for the PCOS group to the control group, we estimated that a minimum of 41 participants were needed (27 in the PCOS group and 14 in the control group).

### 2.6 Statistical methods

All statistical analyses were performed using the SPSS software (version 26.0; SPSS Inc., Chicago, IL, USA). Count data were analyzed using the chi-squared test. Kolmogorov-Smirnov statistics were used to test the normality of the measurement data. Variables with a normal distribution were expressed as mean ± standard deviation, and between-group comparisons were performed using an independent samples t-test. Non-normally distributed variables are presented as medians (25^th^ and 75^th^ percentiles), and between-group comparisons were performed using the Mann-Whitney U test. Spearman’s correlation analysis was used to verify correlations. Logistic regression was used to analyze risk factors. *P*-values<0.05 (*P*<0.05) were considered to indicate statistically significant differences.

## 3 Results

### 3.1 Patients screening process

A total of 126 PCOS patients based on the Rotterdam 2003 criteria were recruited from an outpatient endocrinology department. During the screening process, eighty-five patients were excluded for definite reasons: nine patients were under 18 years old; six patients were postmenopausal; seven patients had a BMI of less than 28 Kg/m^2^; five PCOS patients were comorbid with other chronic liver diseases; fifty-two patients with PCOS had not been diagnosed for the first time or had begun drug therapy; six patients declined to participate. Then, forty-one PCOS patients with obesity who met the inclusion criteria were enrolled in the study.

### 3.2 Comparison of the prevalence of NAFLD between the two groups

The prevalence of NAFLD in the obese PCOS and control groups was 75.61% (31/41) and 45.00% (9/20), respectively (χ2 = 5.579, *P*=0.018).

### 3.3 Comparison of general information, body composition, CAP, biochemical indexes, and sex hormone levels between the two groups

Compared to the control group, the obese PCOS group had higher levels of ALT, AST, CAP, TG, TC, LDL-C, TT, FAI, FIns, and HOMA-IR, and lower levels of HDL-C and SHBG (*P <*0.05). There were no statistically significant differences in age, BMI, waist circumference, WHR, body fat percentage, VFA, or FBG between the two groups (*P* > 0.05). These differences are shown in **Table 1**.

**Table 1:** Comparison of general information, body composition, CAP, biochemical indexes and sex hormone levels between control group and obese PCOS group.

	Control (n=20)	Obese PCOS (n=41)	*t(Z)*value	*P* value
Age	26.05 ± 6.66	25.73 ± 5.64	0.195	0.846
BMI (Kg/m^2^)	30.49 ± 2.77	31.99 ± 4.16	1.676	0.100
WHR	0.95 ± 0.03	0.96 ± 0.05	1.010	0.317
Body fat percentage (%)	42.67 ± 5.11	43.55 ± 5.01	0.630	0.525
VFA (cm^2^)	176.68 ± 35.53	179.76 ± 34.51	0.323	0.748
CAP (db/m)	286.10 ± 23.80	307.90 ± 24.88	3.258	0.002 ^*^
ALT (U/L)	24.50 (15.25,47.25)	41.00 (26.50,62.00)	-2.828 ^a^	0.005 ^*^
AST (U/L)	18.14 (14.50,23.75)	23.00 (18.00,36.50)	-2.585 ^a^	0.010 ^*^
FBG (mmol/L)	5.09 ± 0.53	5.75 ± 0.68	3.823	0.665
TG (mmol/L)	1.66 ± 0.38	2.98 + 1.07	7.040	<0.001 ^*^
TC (mmol/L)	4.25 ± 0.57	4.90 ± 0.96	2.831	0.006 ^*^
HDL (mmol/L)	1.22 ± 0.24	1.08 ± 0.20	2.371	0.021 ^*^
LDL (mmol/L)	2.62 ± 0.58	3.25 ± 0.86	2.951	0.005 ^*^
TT (nmol/L)	2.16 ± 0.56	3.05 ± 0.83	4.378	<0.001 ^*^
SHBG (nmol/L)	31.90 (25.80,40.02)	16.70 (12.30,18.80)	-5.440 ^a^	<0.001 ^*^
FAI (%)	6.53 (5.36,8.78)	20.78 (13.85,26.31)	-5.055 ^a^	<0.001 ^*^
FIns (mU/L)	15.65 ± 2.93	23.22 ± 10.64	4.237	<0.001 ^*^
HOMA-IR	3.41 (2.94,4.01)	5.72 (3.41,7.34)	-3.365	0.001 ^*^

PCOS, polycystic ovary syndrome; BMI, body mass index; WHR, waist-to-hip ratio; VFA, visceral fat area; CAP, controlled attenuation parameter; ALT, alanine transaminase; AST, aspartate transaminase; FBG, fasting blood glucose; TG, triglyceride; TC, total cholesterol; LDL-C, low-density lipoprotein cholesterol; HDL-C, high-density lipoprotein cholesterol; TT, totle testosterone; SHBG, sex hormone binding globulin; FAI, free androgen index; FIns, fasting insulin; HOMA-IR, homeostasis model assessment-insulin resistance.

Results are presented as mean ± standard deviation or medians (25th and 75th percentiles).

^*^: vs. Control group, P < 0.05; ^a^: Z value.

### 3.4 Correlation between liver CAP and differential indicators in PCOS patients with obesity

Liver CAP was positively correlated with ALT, AST, TG, TT, FAI, FIns, and HOMA-IR (*P* < 0.05), and negatively correlated with SHBG (*P* < 0.05). There were no significant correlations between liver CAP and TC, LDL-C, or HDL-C levels (*P >*0.05). The results of correlation analysis are shown in **Table 2**.

**Table 2:** Analysis of indicators with correlation to CAP in PCOS patients with obesity.

		ALT	AST	TG	TC	LDL-C	HDL-C	TT	SHBG	FAI	FIns	HOMA-IR
CAP	*r_s_* Value	0.449	0.387	0.428	0.048	-0.048	-0.148	0.476	-0.468	0.543	0.751	0.688
	*P* Value	0.003	0.012	0.005	0.766	0.764	0.357	0.002	0.002	<0.001	<0.001	<0.001

CAP, controlled attenuation parameter; ALT, alanine transaminase; AST, aspartate transaminase; TG, triglyceride; TC, total cholesterol; LDL-C, low-density lipoprotein cholesterol; HDL-C, high-density lipoprotein cholesterol; TT, totle testosterone; SHBG, sex hormone binding globulin; FAI, free androgen index; FIns, fasting insulin; HOMA-IR, homeostasis model assessment-insulin resistance.

### 3.5 Analysis of risk factors for NAFLD in PCOS patients with obesity

The presence of NAFLD (defined as NAFLD with CAP ≥ 244 db/m) in PCOS patients with obesity was selected as the dependent variable. TG, TT, SHBG, FAI, FIns, and HOMA-IR, which were statistically significant in the correlation analysis, were selected as independent variables. The results of univariate logistic regression suggested that TG, TT, FIns, and HOMA-IR were risk factors for NAFLD in PCOS patients with obesity. Variables with statistical significance in the univariate analysis were further analyzed using multivariate logistic regression. The results revealed that TT was an independent risk factor for NAFLD in obese patients with PCOS. The results of risk factor analysis are shown in **Table 3**.

**Table 3:** Analysis of risk factors for NAFLD in PCOS patients with obesity.

	univariate logistic regression			multivariate logistic regression		
	OR	95% CI	*P* Value	OR	95% CI	*P* Value
TG	3.287	1.114-9.700	0.031	1.205	0.247-5.885	0.818
TT	7.655	1.741-33.666	0.007	11.010	1.239-97.844	0.031
FIns	1.169	1.031-1.326	0.015	1.725	0.826-3.602	0.329
HOMA-IR	1.587	1.037-2.429	0.033	0.243	0.024-2.421	0.228
SHBG	0.857	0.754-0.974	0.018			
FAI	1.065	0.982-1.155	0.126			

TG, triglyceride; TT, totle testosterone;FIns, fasting insulin; HOMA-IR, homeostasis model assessment-insulin resistance; SHBG, sex hormone binding globulin; FAI, free androgen index; CI, confidence interval.

## 4 Discussion

This cross-sectional study aimed to investigate the prevalence and risk factors of NAFLD in PCOS patients with obesity by analyzing the changes in liver CAP in PCOS patients with obesity and the factors that influence it. To our knowledge, this is the first study to enroll obese Chinese women with PCOS as subjects for the FibroTouch test, and is further the first study with a weight- and age-matched control group included to observe and analyze changes in liver CAP. Our findings further showed that the prevalence of NAFLD was significantly higher in the obese PCOS group than that in the non-PCOS group (75.61% vs. 45.00%). Liver steatosis, transaminase levels, sex hormone disorders, and glucolipid metabolism were all found to be more severe in PCOS patients with obesity, as evidenced by the significant elevation in CAP, ALT, AST, TG, TC, LDL-C, TT, FAI, FIns, and HOMA-IR, and the significant decrease in HDL-C and SHBG levels in PCOS patients with obesity. Further analysis showed that hypertriglyceridaemia, HA, and IR were associated with elevated liver CAP in PCOS patients with obesity, while TT was an independent risk factor for the prevalence of NAFLD in PCOS patients with obesity.

PCOS can result in several metabolic disorders, and NAFLD is closely related to metabolic syndrome. Prior research has also indicated that NAFLD is the hepatic manifestation of metabolic syndrome; therefore, there is a close relationship between PCOS and NAFLD (21–23). In a previous study, the prevalence of NAFLD in PCOS, using the Rotterdam criteria as diagnostic criteria, was distributed between 32.9% and 77.0% (24). In the present study, the prevalence of NAFLD in PCOS patients with obesity was 75.61%, and the levels of liver CAP as well as ALT and AST activities in PCOS patients with obesity were significantly higher than those in controls, which further confirms that PCOS could exacerbate hepatic steatosis and liver function damage. These results suggest that liver CAP has important implications for investigating the factors affecting hepatic steatosis in obese patients with PCOS.

It is well known that obesity can lead to NAFLD (25). Therefore, in the present study, we enrolled an age- and BMI-matched control group with no statistical difference in BMI, WHR, body fat percentage, or VFA to the experimental group, thereby reducing the bias in the results potentially caused by obesity. The results showed that, compared to the control group, the obese PCOS group had more severe disorders of lipid metabolism (TG, TC, LDL-C, HDL-C), insulin resistance (FIns, HOMA-IR), and sex hormone disorders (TT, FAI, SHBG), further confirming that there is a correlation between PCOS and endocrine and metabolic disorders.

Similar to our results which showed higher TG, TC, and LDL-C levels and lower HDL-C levels in obese patients with PCOS, a previous study by Liu et al. noted the presence of an abnormal lipid pattern in patients with PCOS (26), including low levels of HDL-C and high levels of TG, TC, and LDL-C. Correlation analysis and univariate logistic regression showed that the TG level was a risk factor for NAFLD in PCOS patients with obesity. Hypertriglyceridaemia induces inflammatory responses and apoptosis in hepatocytes, thereby increasing both the susceptibility to and severity of NAFLD (27). Lipotoxic hepatocyte-derived extracellular vesicle-encapsulated miR-9-5p downregulated the expression of transglutaminase2 and promoted M1 polarisation in macrophages, thereby facilitating NAFLD progression (28). Hypertriglyceridemia also affected pancreatic α and β cells, leading to hyperinsulinaemia and IR (29), further aggravating metabolic disorders and promoting NAFLD.

IR occurs in approximately 75% of PCOS patients, with a higher proportion of IR in PCOS patients with obesity, and IR interacts with PCOS to worsen this situation (30, 31). Overall, our results showed that hyperinsulinaemia and IR were more pronounced and were risk factors for NAFLD in PCOS patients with obesity, consistent with the findings of Zhang et al. (32). Reduced insulin sensitivity of peripheral tissues and diminished inhibition of lipolysis by insulin in IR leads to ectopic deposition of excess free fatty acids in the liver (33). Furthermore, IR promotes *de novo* hepatic lipogenesis (34), increased lipotoxicity, oxidative stress, and inflammatory cascade activation, leading to liver damage (35). IR also activates hepatic stellate cells and contributes to hepatic fibrogenesis (36). Gangale et al. showed that patients with PCOS treated with the insulin sensitizer metformin had improved IR and reduced ALT levels (37). Riemann et al. demonstrated that metformin reduced the hepatic steatosis index in PCOS patients (38). This suggests that insulin sensitizers may provide an important future direction for the treatment of NAFLD in PCOS patients with obesity.

HA is an important clinical feature in patients with PCOS. The pulse frequency of gonadotropin-releasing hormone at sustained high frequencies in PCOS patients causes an increase in luteinizing hormone (LH) pulse amplitude, an overproduction of LH, and a relative lack of follicle-stimulating hormone, resulting in HA (39). Increases in TT and FAI levels were observed in PCOS patients with obesity, with a simultaneous decline in SHBG levels in our study. SHBG binds androgens with high affinity (40), and a reduction in SHBG can further worsen HA. Previous studies have shown that HA is an independent risk factor and predictor of NAFLD in patients with PCOS (41, 42), while PCOS patients with HA have more pronounced hepatic steatosis than those without HA (43). Similar results were observed in the present study. HA promotes the development and progression of NAFLD by increasing hepatic fatty acid *de novo* synthesis (44), inducing apoptosis and autophagy imbalance (45), impairing hepatic branched-chain amino acid metabolism (46), inducing mitochondrial β-oxidation imbalance, and upregulating the expression of inflammatory factors in hepatocytes (47). Overall, our results suggest that liver conditions should be systematically assessed in PCOS patients with obesity with HA, as improvement of HA not only restores reproductive and endocrine disorders in patients with PCOS, but also contributes to improved liver function.

Nevertheless, this study has several limitations which should be noted. First, this was a single-center cross-sectional study with a small sample size and a relatively limited number of patients, which may have contributed to the difference in the results between univariate logistic regression and multivariate logistic regression. There might be some instability in this result. Second, this study did not capture information on the patients’ dietary structure, small intake of alcohol, exercise, and other lifestyle factors, which could potentially influence the patients’ liver steatosis and affect the accuracy of the results. Future studies should validate the effects of liver CAP and risk factors for NAFLD in PCOS patients with obesity by conducting multicenter, large-sample-size studies based on the inclusion of influencing factors, such as diet and lifestyle.

## 5 Conclusion

In summary, PCOS patients with obesity had a significantly higher prevalence of NAFLD and liver CAP and transaminase levels, suggesting a more pronounced hepatic steatosis and liver damage. Hypertriglyceridaemia, IR, and HA were associated with changes in liver CAP in PCOS patients with obesity, and TT was an independent risk factor for NAFLD in PCOS patients with obesity.

## Data availability statement

The original contributions presented in the study are included in the article/supplementary material. Further inquiries can be directed to the corresponding author.

## Ethics statement

This study was audited by Medical Ethics Committee of Shengjing Hospital of China Medical University (Audite No. 2021PS647K). The studies were conducted in accordance with the local legislation and institutional requirements. The participants provided their written informed consent to participate in this study.

## Author contributions

DW and BH conceived, designed, and performed the research. NN and HB conducted the FibroTouch examination for the PCOS patients and control subjects. DW wrote the paper. BH reviewed and edited the fifinal manuscript. All authors read and approved the final manuscript. All authors contributed to the article and approved the submitted version.
